# Cardiovascular Risk Factors in Children with Obesity, Preventive Diagnostics and Possible Interventions

**DOI:** 10.3390/metabo11080551

**Published:** 2021-08-20

**Authors:** Mirjam Močnik, Nataša Marčun Varda

**Affiliations:** 1 Department of Paediatrics, University Medical Centre Maribor, Ljubljanska ulica 5, 2000 Maribor, Slovenia; natasa.marcunvarda@siol.net; 2 Medical Faculty, University of Maribor, Taborska 8, 2000 Maribor, Slovenia

**Keywords:** obesity, children, cardiovascular risk, hypertension, diagnostics, prevention

## Abstract

The increasing burden of obesity plays an essential role in increased cardiovascular morbidity and mortality. The effects of obesity on the cardiovascular system have also been demonstrated in childhood, where prevention is even more important. Obesity is associated with hormonal changes and vascular dysfunction, which eventually lead to hypertension, hyperinsulinemia, chronic kidney disease, dyslipidemia and cardiac dysfunction—all associated with increased cardiovascular risk, leading to potential cardiovascular events in early adulthood. Several preventive strategies are being implemented to reduce the cardiovascular burden in children. This paper presents a comprehensive review of obesity-associated cardiovascular morbidity with the preventive diagnostic workup at our hospital and possible interventions in children.

## 1. Introduction

In recent decades, the prevalence of obesity has increased worldwide and has become a frequently discussed topic. However, it is still being researched in detail due to its complexity and numerous consequences. Since 1975, the prevalence of obesity has nearly quadrupled among children and adolescents with an increased likelihood of obesity in adulthood [1,2]. In our country, a study conducted in 2012/2013 showed that 12.7% of schoolchildren were overweight, with 8.4% in the overweight and 4.3% in the obese category [3]. Along with increasing obesity, associated comorbidities, such as obesity-related hypertension, cardiac dysfunction, hyperinsulinemia, dyslipidemia and chronic kidney damage, are being increasingly recognized [4]. All of the abovementioned can lead to an increased frequency of cardiovascular diseases in the future that have been recognized as the leading cause of mortality throughout the world. According to the World Health Organization, ischemic heart disease and stroke are the leading causes of death globally [5].

The common denominator of all cardiovascular diseases is atherosclerosis, which begins in childhood [6]. Early atherosclerotic changes have been found in autopsied infants [7]. In addition, autopsied adolescents and young adults aged 15–34 years who died as a result of an accident, homicide or suicide were shown to have a positive association of atherosclerosis in the coronary arteries and abdominal aorta with elevated non-HDL cholesterol, hypertension, impaired glucose tolerance and obesity, and a negative association with the HDL cholesterol concentration [6], all considered to be classic risk factors for cardiovascular events. The data also suggest an earlier incidence of cardiovascular complications in at-risk groups of children later in adulthood [8,9].

Adipose tissue, especially visceral obesity, is thought to induce a variety of alterations in cardiovascular structure and function [10]. In this paper, we present a review of the development of obesity-related cardiovascular risk factors with possible early diagnosis and interventions.

## 2. Pathophysiology of Vascular Dysfunction in Obesity

**Figure 1:**
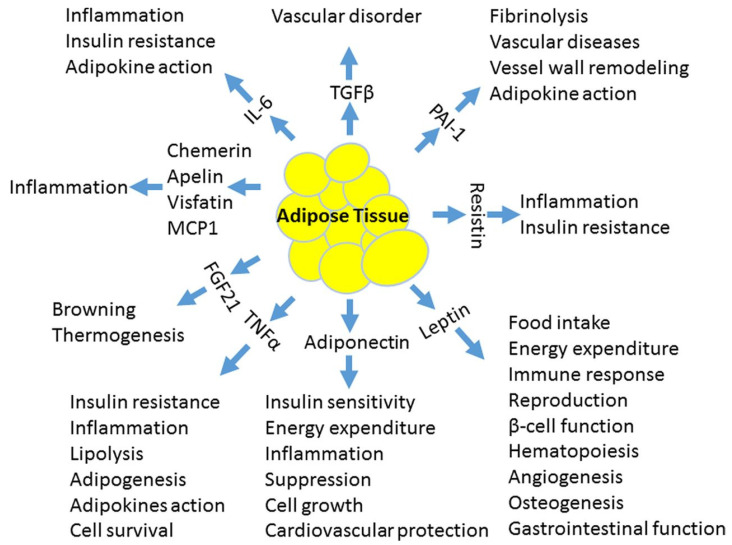
Several highly active molecules are released from adipocytes and affect vascular endothelial function by modulating the balance between nitric oxide synthesis and reactive oxygen radicals. Adapted by Luo et al. [15]. PAI-1, plasminogen activator inhibitor-1; MCP-1, monocyte chemoattractant protein-1; IL-6, interleukin-6; TNF-alpha, tumor necrosis factor-alpha; FGF21, fibroblast growth factor 21; TGF-beta, transforming growth factor beta.

Historical perception of adipose tissue as a storage site of fatty acids has been replaced over recent decades by the notion that adipose tissue acts as an active endocrine organ that is closely involved in the production of proatherogenic adipokines, oxidative stress and chronic inflammation [11]. The presence of obesity alone is a risk for endothelial dysfunction [12]. The term “endothelial dysfunction” describes the functional state of the vascular endothelium switching from a healthy atheroprotective monolayer to a layer of cells expressing a proatherosclerotic profile. These actions are due to the (in)balance of substances released by endothelial cells [13]. Under normal circumstances, vasodilators (nitric oxide (NO), endothelial-derived hyperpolarizing factor and prostacyclin) and vasoconstrictors (prostaglandin, endothelin-1 and angiotensin-II) are in balance to provide normal vascular function. However, in obesity, this delicate balance, influenced by a large number of molecules, is usually disrupted, leading to platelet activation, leucocyte adherence, vasoconstriction, pro-oxidation, vascular inflammation, impaired coagulation, atherosclerosis and thrombosis with subsequent cardiovascular damage [14,15]. The release of some highly active molecules from adipose tissue is schematically summarized in Figure 1 [14].

**Figure 2:**
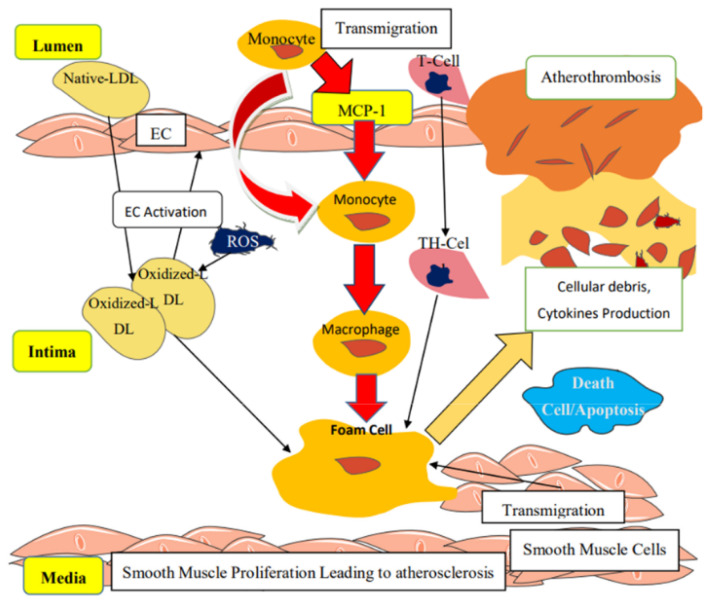
The process of atherosclerosis, adapted by Kwaifa et al. [14]. LDL, low-density lipoprotein; EC, endothelial cell; ROS, reactive oxygen species; TH, thrombocyte; MCP-1, monocyte chemoattractant protein-1.

An imbalance between nitric oxide synthesis and reactive oxygen radicals leads to endothelial damage, secretion of cytokines, chemokines and adhesion molecules. Monocyte chemoattractant protein-1 (MCP-1), in particular, attracts leucocytes from the blood. Next, leucocytes are attached to the endothelium with adhesion molecules. Transmigration to the subendothelium, differentiation and maturation to macrophages follows. The macrophages then take up accumulated oxidized (by reactive oxygen radicals) low-density-cholesterol lipoproteins, resulting in foam-cell formation. Along with these processes, smooth muscle cells proliferate and migrate into the subendothelial space, causing fibrous cap formation with the increased extracellular matrix, resulting in thickening of the intima with subsequent formation of the atherosclerotic plaque [14]. The process is schematically shown in Figure 2 [14].

Several hormones and cytokines—with the most commonly researched being leptin, adiponectin, ghrelin, resistin, tumor necrosis factor (TNF)-α, interleukin (IL)-6, IL-10, IL-1β, MCP-1, plasminogen activator inhibitor (PAI)-1 and proteins of the renin–angiotensin system (RAS)—are released inappropriately in obesity and have been implicated in the development of endothelial dysfunction [14,15,16]. Therefore, obesity increases lipid peroxidation and induces persistent platelet activation, affecting the vascular endothelial function and probably leading to premature atherogenicity [17].

Leptin is a hormone predominately produced in adipose cells that mainly functions to regulate energy balance by inhibiting hunger [18]. However, studies have shown that in obesity consequential hyperleptinemia reduces NO bioavailability by reducing endothelial NO synthase [19]. In obesity, adiponectin and ghrelin levels are decreased [18]. Adiponectin acts as a glucose and fatty acids regulator with increased levels in food restriction and decreased levels in obesity. It enhances glucose utilization; however, its function is still not completely understood. It is believed to affect the 5′ adenosine monophosphate-activated protein kinase and cyclooxygenase-2 pathways with a reduction in cell apoptosis, promotion of NO production, a decrease in TNF-α activity and prevention of atherosclerotic proliferation. Due to its decreased concentration in obesity, these pathways are disrupted, thus promoting vascular dysfunction. This has also been proven by alleviation of vascular dysfunction in animal models when adiponectin is added [20,21,22]. Ghrelin is another hormone that is decreased in obesity, since its function is to increase food intake. Its link to the vascular system has been demonstrated in experimental and clinical studies. It has been shown that ghrelin reverses endothelial dysfunction with an increase of NO availability; hence, its lack in obesity could contribute to the promotion of atherosclerosis [23,24,25]. Resistin is produced not only in adipocytes but also in mononuclear leucocytes, macrophages, spleen and bone marrow, which suggests that resistin is linked to inflammation. Its level increases with obesity, with central obesity in particular, contributing to rising levels of resistin. Physiologically, it has a role in glucose metabolism and insulin resistance. Its vascular dysfunction is promoted by increased endothelin-1 release and decreased NO bioavailability [26,27,28].

Nowadays, obesity is perceived as a chronic inflammatory state. With excessive food intake, there is an increased demand for lipid storage. Consequently, adipose tissue hyperplasia and hypertrophy lead to chronic inflammation with the altered secretory output of inflammatory markers [14,29]. It has been shown that several inflammatory markers are increased in obesity and are believed to be critical determinants of pathological alterations to the vasculature [29]. The increased levels of IL-6 and TNF-α in obesity could also affect endothelial NO synthase expression [19]. IL-1β increases the expression of cell adhesion molecules and MCP-1 affects smooth muscle cells in vessel walls. Next, IL-4 increases the generation of reactive oxygen species and induces proliferation and migration in vascular smooth muscle cells [28]. In contrast, IL-10 is believed to be an anti-inflammatory cytokine with inhibition of TNF-α, IL-1β, IL-6 and IL-8. It also increases NO bioavailability. In children with obesity, its level is reduced and its expression downregulated [29,30].

The early endothelial damage associated with obesity is confirmed by the high levels of von Willebrand factor, D-dimer concentration, thrombin–antithrombin complex, PAI-1 and fibrinogen [17]. PAI-1 inhibits tissue plasminogen activator and urokinase, the activators of plasminogen and, hence, fibrinolysis. Therefore, elevated PAI-1 is a risk factor for thrombosis. In children with obesity, its level is markedly increased suggesting that even in the young, obesity is associated with a potential prothrombotic state [31,32]. PAI-1, which has also been found to be elevated in young males after myocardial infarction, serves as a novel marker for cardiovascular risk, particularly in young males with obesity and insulin resistance [32].

Renin and angiotensin also seem to play an important role in vascular dysfunction, eventually promoting hypertension. In humans and animal models of obesity, activation of the renin–angiotensin–aldosterone system (RAAS) has been observed to be associated with enhanced oxidative stress and inflammation in the vascular tissue [33]. The effect is mediated through inappropriate mineralocorticoid receptor activation in adipose tissue [34].

## 3. Obesity and Hypertension in Children

Hypertension in children was historically a rare disease, most commonly associated with secondary causes (renovascular, hormonal, etc.); however, largely due to the obesity epidemic, essential hypertension has become one of the most common childhood illnesses in the last decade [35]. In a cohort of participants in our study to determine the prevalence of pre-hypertension and hypertension, overweight participants had a 1.75 greater relative risk of pre-hypertensive blood pressure and obesity carried a 1.79 times greater relative risk of blood pressure outside of the normotensive blood-pressure range [36]. The latter is defined as systolic and diastolic blood pressure below the 90th percentile for age, gender and height, while hypertension is defined as values above the 95th percentile. Blood pressure should be measured on at least three separate occasions or confirmed by ambulatory blood-pressure measurement [35,37].

The pathophysiology of hypertension in obesity is associated with various pathophysiological mechanisms, namely with vascular damage as a consequence of inflammation, endothelial dysfunction and oxidative stress, with sympathetic activation due to elevated leptin and insulin levels, with vasoconstriction, sodium and fluid retention through activation of RAAS and with decreased vagal activity [38,39]. Some details of the pathophysiology of vascular dysfunction are discussed above.

Studies in adults have shown that the risk of hypertension increases with increasing body mass index [38]. In adults, the risk of having obesity-related hypertension is even higher, e.g., a 3.5-times increased likelihood of having hypertension with obesity [39], which increases the likelihood of obese children having hypertension early in adult life if not already in childhood. It has been estimated that 60–70% of hypertension in adults may be directly attributable to adiposity [40].

## 4. Obesity and Cardiac Dysfunction in Children

Obesity has been traditionally associated with cardiac dysfunction, especially with increased left ventricle stress, compensatory left ventricle hypertrophy and abnormal diastolic function without consistent systolic dysfunction [41,42,43]. These changes are thought to be mostly due to obesity-associated comorbidities; however, even obesity itself can affect cardiac function in the absence of hypertension with significant impairment of longitudinal myocardial deformation properties [44]. In later studies, a different and independent impact of obesity and hypertension on cardiac dysfunction in children was demonstrated, with worsening diastolic function in obesity, and hypertrophic concentric remodeling of the left ventricle in hypertension, which was not found in previous studies [45]. Additionally, recent findings suggest that hepatic steatosis is related to early atherosclerosis and cardiac dysfunction, with an increase in left ventricular mass, systolic and diastolic dysfunction, and increased epicardial adipose tissue thickness, even in the pediatric population [46].

These subclinical manifestations of cardiac function are important because, with treatment, the process can still be reversed and is probably more effective earlier in the disease [44,47].

## 5. Obesity and Chronic Kidney Disease in Children

Obesity affects kidney function and has been recognized as one of the strongest risk factors for new-onset chronic kidney disease (CKD). In obesity, compensatory hyperfiltration occurs to meet the heightened metabolic demands of the increased body weight. Consequently, intraglomerular pressure is higher and this can damage the kidneys leading to long-term kidney damage and CKD [48]. Additionally, inappropriate levels of adipokines may be factors in the development of CKD, with obesity-related comorbidities (e.g., hypertension, dyslipidemia and insulin resistance), further influencing the development of CKD in obese adults and children. Specifically, altered levels of adipokines can increase glomerular permeability, fuse the podocytes and cause mesangial cell hypertrophy, all of which can alter the glomerular filtration rate [49,50].

Non-alcoholic fatty liver disease (NAFLD), caused by an accumulation of fat in the liver in children with obesity, has been associated with an increased prevalence of CKD independently of traditional cardiorenal risk factors. The proposed mechanism is exacerbated insulin resistance, which predisposes to atherogenic dyslipidemia and further vascular damage, most obviously affecting kidney function. However, a causal association has not been definitely established [51].

There is a reciprocal effect between cardiovascular risk factors and CKD: cardiovascular risk factors accelerate kidney damage and CKD is a risk factor for cardiovascular events. Early markers of atherosclerosis with increased arterial stiffness and intima-media thickness are frequently present in children with CKD, especially those on dialysis. Therefore, early CKD, before dialysis is required, is the optimal time for risk factor identification and intervention to prevent further kidney damage and possibly cardiovascular events in the future [52,53]. The earliest consequence of obesity-related CKD in the general population is usually albuminuria without kidney failure, demonstrating a window of opportunity for early intervention [54].

## 6. Obesity and Dyslipidemia in Children

Dyslipidemia is commonly associated with obesity, mainly driven by insulin resistance and alterations in adipokines. In recent years, a distinct subgroup of dyslipidemia in obesity has been established, the so-called metabolic dyslipidemia, with high concentrations of triglycerides and decreased high-density lipoprotein cholesterol (HDL), while low-density lipoprotein cholesterol (LDL) is only mildly increased. Metabolic dyslipidemia is associated with a proatherogenic state. It has been suggested that proatherogenity might not be as prominent in obese subjects with a healthy lipid profile. Metabolic dyslipidemia is more often present in visceral obesity and is exaggerated by alteration to adipokines. Recent investigations have been further extended to epigenetic mechanisms that may be implicated in the regulation of obesity phenotypes, with an emphasis on studies on microRNAs expression [55].

The Bogalusa Heart Study established that schoolchildren with overweight were 2.4 to 7.1 times more likely to have elevated total cholesterol, LDL and triglycerides in comparison to thin peers [56]. Almost half of the children with obesity have a type of dyslipidemia [57]. According to some studies, hypertriglyceridemia is more often present [57], while in others, lower HDL predominates [58]. The ratio between apolipoprotein B and A1 has been shown to be a good predictor of cardiovascular disease [59]. Small, dense low-density lipoproteins (sdLDL) are also being studied as independent emerging cardiovascular risk factors that are often elevated in obese individuals [60].

## 7. Obesity and Hyperinsulinemia in Children

Insulin resistance is characterized by the impairment of insulin action, resulting in persistent hyperglycemia and increased insulin production, e.g., hyperinsulinemia, frequently observed in children with obesity [61,62]. Hyperinsulinemia can be present without significant insulin resistance and is an important independent predictor of type 2 diabetes [63]. Hyperinsulinemia and insulin resistance should therefore be considered independently, even though they are closely intertwined and usually coexist [64].

Excess fat, especially visceral fat, has been considered the main cause of insulin resistance. The proposed mechanism consists of impaired insulin signaling pathways in dysfunctional endothelial cells leading to blunted vasodilatation, abnormal capillary recruitment and impaired substrate delivery by insulin to target tissues [65]. Insulin resistance can induce an imbalance in glucose metabolism, leading to chronic hyperglycemia, which in turn triggers oxidative stress and causes inflammatory damage. Insulin resistance can also alter lipid metabolism leading to the so-called metabolic dyslipidemia [66]. However, the pathophysiological mechanism has not been fully explained and proven with experimental data. Therefore, an interesting hypothesis is emerging originating from the assumption that hyperinsulinemia is the initiating defect leading to increased nutrient consumption and hyperlipidemia. The cause of hyperinsulinemia is presumed to include food additives, which have entered our food supply in recent decades. Hyperinsulinemia is also sustained by hyperlipidemia from the increased adipose mass and leads to insulin resistance [62]. In one study, higher body mass index preceded hyperinsulinemia, thus undermining the stated theory [67]. Further research is needed to confirm any of these hypotheses.

Higher body mass index has been found to be associated with hyperinsulinemia even in childhood, confirming its significant role in the development of metabolic syndrome and type 2 diabetes mellitus in adult life [67]. The latter is traditionally associated with increased cardiovascular morbidity and mortality in adulthood. Recent studies suggest that controlled glycemia alone is not essential for decreasing cardiovascular risk. More importantly, improving insulin resistance has shown a lessening of diabetes complications, including cardiovascular events. It has been estimated that insulin resistance is the most important single cause of coronary artery disease [68]. In prepubertal children, glucose and insulin metabolism have been found to be associated with adult cardiovascular risk and markers of atherosclerosis [69].

## 8. Evaluation and Possible Interventions in Obesity-Related Cardiovascular Risk Factors in Children

Although cardiovascular disease is rare in children, risk factors for cardiovascular disease are significantly more common. In modern society, they are on the rise due to the increasing prevalence of obesity. Together, they accelerate childhood atherosclerosis. Therefore, preventive measures are essential and also most effective in children and young adults. There is growing evidence that preventive activities can slow down the atherosclerotic process and delay the clinical manifestation of cardiovascular disease [70].

Preventive measures include two complementary approaches: the high-risk approach and the population approach. The high-risk approach is focused on finding and treating individuals who are classified as high cardiovascular risk; therefore, this is an important preventative activity. At-risk children are those who, due to an underlying disease, inappropriate lifestyle or genetic predisposition, are more likely to develop early cardiovascular ageing and complications later in life. The population approach considers the entire population and is aimed at a favorable shift of cardiovascular risk factors towards lower levels. It is interventionally and educationally oriented and effective at the population level [71].

At the Maribor University Hospital, the Unit for Paediatric Nephrology and Arterial Hypertension, a high-risk approach is being consistently implemented. Children with obesity are evaluated by using the investigations presented in Table 1.

The management of a child with cardiovascular risk factors includes a good history, clinical examination, and laboratory and imaging tests to assess the presence and extent of a particular risk factor and to determine necessary investigations. We focus on a family history of cardiovascular factors (e.g., cardiovascular event before the age of 50 years), on information about possible symptoms due to target organ damage or secondary causes (secondary hypertension, hormonal diseases, etc.), assess physical activity and eating habits, psychosocial condition and possible tobacco, alcohol or drug abuse. In the clinical examination, we perform anthropometric measurements, which are evaluated with reference to percentile curves. If random blood-pressure measurements are found to be elevated, 24-h blood-pressure measurement is also performed [73].

Basic laboratory tests comprise renal function markers, including cystatin C, and markers of liver damage (fatty liver infiltration), together with electrolytes, urate, blood sugar and the lipid profile [73]. Urate is considered an independent indicator of arterial hypertension in children [74] and is also associated with renal dysfunction, albuminuria and obesity in childhood [75]. In obesity, the basic lipid profile and additional lipoproteins, such as apolipoprotein A1 and B, which are considered additional indicators of dyslipidemia and an additional risk factor for accelerated atherosclerosis in the pediatric population, are determined [76]. In recent years, there have been new insights into metabolic dyslipidemia, seen in the obese and discussed above, in recent years. The ratio between triglycerides and HDL cholesterol has been confirmed to be a useful marker of cardiovascular risk as an independent determinant of arterial stiffness, especially in obese youth [77,78]. Lipoprotein (a) (Lp(a)) was not found to be solely associated with obesity but is regarded as an independent cardiovascular risk factor. Lp(a) has been found to be high in children with a family history of premature cardiovascular events. In children with obesity, elevated Lp(a) presents an additional risk factor [79]. Elevated levels of homocysteine have been found in children with abdominal obesity together with hypertension and dyslipidemia and may indicate a high-risk constellation that should be monitored [80,81].

Vitamin D is being checked routinely since its deficiency is now recognized as a pandemic [82] observed more frequently in obese children and adolescents [83]. Its deficiency has also been associated with insulin resistance [83] and elevated blood pressure [84]. In children with obesity and hepatic steatosis, vitamin D levels were significantly lower than in children with obesity without hepatic steatosis [85]. Some studies have shown an association between vitamin D deficiency and a higher risk of cardiovascular disease and mortality, which indicates the need for its supplementation [86].

Additionally, several new biomarkers are emerging as indicators of cardiovascular risk, such as kidney injury molecule 1 (KIM-1), adropin, salusin-α and -β, uromodulin, and markers of oxidative stress, and these are currently being investigated at our department but are not routinely used. KIM-1 is already a known marker of acute tubular necrosis, and its level is also increased in overweight children [87,88]. Salusin-α and -β are considered biomarkers for coronary heart disease and arterial hypertension. Salusin-β acts proatherogenically to stimulate the formation of macrophage foam cells, which stimulate the inflammatory response of endothelial cells. Salusin-α inhibits the formation of macrophage foam cells and subdues the inflammatory response of endothelial cells. Research has shown that these molecules have an opposing effect on the pathogenesis of atherosclerosis. Research to date suggests that the salusin-α level is decreased and the salusin-β level is increased in adult patients with hypertension [89]. In children, salusin-α levels are negatively correlated with diastolic pressure [90]. Adropin serves as a novel regulator of endothelial function. Its function is broad, including angiogenesis and the metabolism of glucose, fatty acids and dyslipidemia. It has a protective role; hence, decreased levels in overweight children [91] suggest its possible involvement in the pathogenesis of obesity-related metabolic comorbidities. Uromodulin has an immunomodulatory role and has been evaluated as a predictive marker in the urine in patients with CKD. Its level in the blood has been shown to be a useful marker in the prognosis of cardiovascular disease in the elderly [92], but little research has been performed in regard to children. In children with diabetes, a lower level correlated negatively with albuminuria [93]. The definition of oxidative stress is an imbalance between free radicals and antioxidant mechanisms, where an excess of free radicals (due to increased production or lack of antioxidant mechanisms) leads to their binding to proteins, lipids and nuclear or mitochondrial genetic material and consequent cell damage. The molecular mechanism of free radicals has not yet been fully elucidated. Research has shown that free radicals are closely associated with endothelial dysfunction [94] and atherosclerosis, which is today considered to be the result of an inflammatory response to endothelial injury [95]. Some research has shown the association between markers of inflammatory stress and overweight in the pediatric population [96,97]. MicroRNA is another possible diagnostic or therapeutic target being increasingly studied. Its function is post-translational. Its role in lipid metabolism has been well recognized, but considerable additional research needs to be performed, especially in the pediatric population [98,99].

In all children, thyroid-stimulating hormone (TSH) is checked since hypothyroidism can be historically associated with weight gain. Additionally, obese children have higher serum TSH and thyroxine levels; however, these levels are still within the normal range. An increase in TSH in the obese is associated with dyslipidemia and higher systolic blood pressure [100].

Albuminuria is considered a reflection of vascular endothelial dysfunction in adult patients and is associated with cardiovascular risk factors in apparently healthy adults and children. However, studies have shown that albuminuria is not strongly associated with cardiovascular risk in children [101,102], and the cardiovascular risk might depend more on the presence of hypertension [103].

To confirm essential hypertension, other secondary causes should be excluded, especially when hypertension persists after non-pharmacological treatment. To exclude primary hyperaldosteronism, the aldosterone-to-renin ratio is determined and evaluated according to age and gender [104]. Next, pheochromocytoma should be excluded as symptoms of catecholamine excess are non-specific. A high index of suspicion should be maintained with sustained hypertension and metanephrines in plasma or a 24-h sample of urine determined [105].

Another possible cause of secondary hypertension is renovascular disease due to renal artery stenosis, most commonly caused by fibromuscular dysplasia in the young and atherosclerotic disease in the elderly. Early diagnosis is important, since interventional therapy may improve or cure hypertension and preserve renal function. Renal artery Doppler ultrasound is the screening test utilized. When appropriate, computed tomography angiography (CTA) and magnetic resonance angiography (MRA) are the next steps in the diagnostic workup. Digital subtraction angiography is reserved for cases with major discrepancies between our intervention and persistence or further elevation of blood pressure [106,107].

Abdominal ultrasound is routinely performed to assess hepatic steatosis in children with obesity. In all children with elevated blood pressure, we also perform an ultrasound of the urinary tract for exclusion of Wilms tumor, neuroblastoma, renal cystic disease, or dysplasia [73,108]. Non-alcoholic fatty liver disease is being increasingly evaluated by using ultrasound elastography in the adult population with some promising results. Ultrasound elastography is an ultrasound technique used to assess the elasticity of tissues. The use of ultrasound elastography opens up a new spectrum of ultrasound applications. In recent years, the use of ultrasonic elastography has spread in various fields, especially in research to assess the degree of liver fibrosis, renal elasticity, the distinction between benign and malignant tumors, musculoskeletal system and other specific areas [109]. The main application of the method is the determination of fibrosis in liver disease of various etiologies. In terms of cardiovascular risk factors, it might be useful in assessing the degree of steatosis or fibrosis in obese patients [110]. Ultrasound elastography might also assess the elastic properties of the kidney. Its use is being researched in the field of chronic renal failure, which has increased in developed countries, due to diabetic and hypertensive nephropathy, which are also the main risk factors for cardiovascular complications. In most cases of chronic renal failure, progression of the disease is associated with an advanced fibrotic process, which may involve the glomeruli or interstitium, depending on the underlying nephropathy. Quantification of intrarenal fibrosis by a non-invasive imaging method could contribute to the overall assessment of renal function [111]. In children, elastic properties of the liver have been successfully obtained [112], while other areas remain the subject of research. In the context of cardiovascular diseases, the predictive value of this method is still unexplored.

Blood vessels can be visualized directly with ultrasound intima-media thickness (IMT) measurement of carotid arteries. It is regarded as a subclinical indicator of atherosclerosis and has a predictive value in the adult population; however, this is less marked in children. Several studies have been performed to assess IMT in association with known cardiovascular risk factors. The association between IMT and obesity [113], familial hypercholesterolemia [114] and hypertension has been demonstrated [115]. Regression of IMT has been shown after normalization of blood pressure and metabolic factors [115]. However, the association between IMT and a family history of premature cardiovascular events has not been proven [113]. Moreover, in some studies, the association is not clear cut and sometimes intra- and interoperative comparability in children raises questions of the usefulness of the method [115].

Another possible ultrasound technique in the evaluation and stratification of the cardiovascular risk profile is the measurement of the anterior–posterior diameter of the abdominal aorta, which has a significant linear relationship with the body mass index in overweight or obese children, indicating initial endothelial dysfunction and vascular damage [116].

Blood vessels can also be evaluated by assessment of arterial stiffness. One of the frequently used methods is pulse wave velocity measurement with applanation tonometry. The pulse wave, created at the systole and transmitted along the arteries with propagation and bouncing at the junctions, carries information about the elasticity of the vessel wall. With higher pulse wave velocity, a less compliant artery is expected, and this is exploited for indirect assessment of subclinical atherosclerosis. It is already a well-researched measurement in children, and it has been proven that a higher pulse wave velocity is characteristic in children with cardiovascular risk factors, e.g., overweight, diabetes mellitus and CKD. The method is still not in routine use due to the time and experience required for qualitative measurements [117,118].

Obesity, frequently in association with arterial hypertension, has a significant impact on the heart, most often presenting with left ventricular hypertrophy. It is usually asymptomatic in children; however, in adults, it is associated with ventricular arrhythmias and heart failure and confers a four-times increased risk of cardiovascular events. In all children with obesity, electrocardiogram (ECG) and echocardiography should be performed to assess left ventricular mass. If left ventricular hypertrophy is present, interventions should be escalated, and follow-up echocardiography performed to demonstrate the regression of hypertrophic tissue [119]. A reduction in myocardial strain has been observed in children with obesity or diabetes mellitus type 2, suggesting that they both have a harmful effect on systolic cardiac function [120]. Additionally, multi-chamber dysfunction with impaired left atrial reservoir function and atrial contractions found in severely obese children suggests an early loss in the compensatory ability of atrial contraction [121].

Both hypertension and diabetes mellitus, which are associated with obesity, are known risk factors for hypertensive and diabetic retinopathy, respectively. Recent studies have shown that retinal vascular changes, defined as arteriovenous crossings or papilledema, can be early complications of overweight and obesity alone, even during childhood and adolescence [122]. A higher body mass index has been shown to be an independent risk factor for diabetic retinopathy, which is accelerated in children with diabetes mellitus type 1 when obesity is also present [123,124]. Therefore, screening for retinopathy with fundoscopic examination should be carried out in all children and adolescents with obesity.

Obesity is followed by hyperinsulinemia and subsequent diabetes mellitus type 2 even in childhood [67]. Detecting hyperinsulinemia with its levels during an oral glucose tolerance test presents a window of opportunity for escalation of treatment. A glycosylated hemoglobin (HbA1c) value of 5.8% is proposed as a screening tool to identify children and adolescents at increased risk [125]. With impaired glucose tolerance and severe hyperinsulinemia, glucose-lowering drugs, such as metformin, may be used to reduce body mass [126,127].

At our hospital, measurement of body composition by using bioelectrical impedance analysis (BIA) is also frequently performed to exclude a potentially increased but not obvious muscle mass and to quantify loss or gain of fat mass with changes in muscle mass. Body composition is closely related to various health outcomes and represents a valuable tool to assess nutritional status [128]. In children, measurement of body composition is important as a predictor of cardiometabolic diseases and can be used in pediatric hypertension risk stratification [129].

Obesity affects several different organ systems throughout the body. Therefore, the problem should be tackled comprehensively. The integral model of cardiovascular disease prevention in childhood is presented in Table 2 [70,71]. We propose close monitoring of children with obesity to detect any evolving additional cardiovascular risk factors that can be treated. Children should be fully evaluated annually according to Table 1, and clinic visits should take place in between to assess bodyweight reduction and blood pressure. Frequent visits allow continuous promotion of a healthy lifestyle and increase compliance. If no success is achieved, specialized obesity-treatment programs should be initiated.

In childhood, primary preventive activity and primordial preventive activity are especially important. Secondary preventive activity includes treatment and measures to treat obesity. It is aimed at early diagnosis and the slowing of weight gain. With the concept of primordial preventive activity, we focus on maintaining an ideal weight in healthy children and continuation in the future. By preventing obesity, we reduce the population burden. In order to implement the concept, it is necessary to establish an environment that promotes health as a value, to ensure state and political support and to include it in a broad-based public health strategy. Primary preventive activities include measures to prevent excessive weight gain before obesity develops and, above all, lifestyle changes [71].

The main treatment for obesity is lifestyle intervention with diet and exercise. Psychological help is often needed to achieve this goal. Children with obesity also face an increased risk of body dissatisfaction, low self-esteem, social isolation and discrimination, and depression; experience a reduced quality of life; and are at risk of ongoing psychosocial distress into early adolescence [130,131]. Psychological complications of obesity include impaired body perception, isolation, aggression, drug and alcohol abuse, bulimia and smoking [132]. There is also an association between obesity and mood and anxiety disorders [133]. Psychologists should be important members of the multidisciplinary team that comprehensively tackles obesity.

Good nutrition, along with a physically active lifestyle and the absence of tobacco use, is essential to delay or prevent the onset of cardiovascular disease. The obesity epidemic has raised the complex issue of matching appropriate energy intake to energy expenditure. In children with a cardiovascular risk factor, a balanced diet with physical activity (60 min of moderate to vigorous play or physical activity daily) is recommended to maintain normal growth. More specific recommendations include eating vegetables and fruit daily with limited juice intake, the use of vegetable oils and soft margarine low in saturated fat and trans-fatty acids, whole-grain bread and cereals, reduction in sugar-sweetened beverages and foods, use of non-fat or low-fat milk and dairy products, the inclusion of oilier fish and the reduction in salt intake and processed foods. In early life, breastfeeding is preferred. Special attention is paid to qualitative nutrients when implementing solid foods, to providing a social context for eating behavior and to continuing education about food and nutrition [134]. When a cardiovascular risk factor is present in a child, the nutritionist can provide additional education and guidance. However, dietetic and behavioral treatments alone can have only limited success. Several pharmacological approaches have been studied. Some weight reduction has been achieved with sibutramine, orlistat, metformin and fluoxetine when it was added to metformin. Pharmacotherapy should be considered only after the failure of intensive lifestyle modification. Sibutramine is a neurotransmitter reuptake inhibitor that acts centrally, enhancing satiety by inhibiting the reuptake of serotonin, norepinephrine and dopamine. Fluoxetine has a similar mechanism of action. Orlistat is a pancreatic lipase inhibitor that reduces fat absorption. Its main adverse effects include gastrointestinal disturbances, such as steatorrhea. Metformin is an antihyperglycemic agent that inhibits hepatic gluconeogenesis and fatty acid oxidation and increases insulin-mediated glucose disposal. All the abovementioned drugs were found to be successful in reducing weight in children with good safety profiles. The use of octreotide, a synthetic analogue of somatostatin, has had some success in children, but it requires parenteral administration. Several other pharmacological interventions are being studied, such as other centrally acting agents, newer lipase inhibitors, β3-adrenoreceptor agonists, glucagon-like peptides, some antiepileptic drugs, endocannabinoid receptor blockers, serotonergic drugs, etc. Their use and safety profile in children need to be investigated [135,136].

## 9. Conclusions

The obesity epidemic in children is contributing significantly to the cardiovascular risk burden that we are facing in the modern world. Obesity-associated cardiovascular diseases and complications involve several organ systems that should be monitored in children with obesity to prevent excessive damage that worsens with continuing obesity in adulthood. We propose frequent monitoring for the possible development of other cardiovascular risk factors and evaluation of end-organ damage to enable timely intervention. We would like to emphasize that, in children with pre-existent cardiovascular risk factors, the natural progression of atherosclerosis can be accelerated, leading to significant cardiovascular events early in life. These should be prevented to facilitate quality and longevity of life. However, primary and primordial prevention are the most important.

## Figures and Tables

**Table 1:** Routine and optional investigations in children with obesity and other possible cardiovascular risk. HDL—high-density lipoprotein, LDL—low-density lipoprotein, TSH—thyroid-stimulating hormone, HbA1c—glycosylated hemoglobin, ECG—electrocardiogram [72].

Investigations	Routine	Optional + Research Level
Laboratory work-up	Blood count, electrolytes, urea, creatinine, urinalysis, total cholesterol, HDL, LDL, triglycerides, markers of liver damage, apolipoprotein A1 and B, lipoprotein(a), urate, homocysteine, cystatin C, TSH, blood sugar, HbA1c and vitamin D; 24-h urine sampling for proteinuria; Albumin/creatinine in morning void sample.	Second-level investigations, e.g., to exclude secondary causes of hypertension, hormonal disorders, etc.; Genetic markers; Novel biomarkers of obesity, chronic kidney disease, etc.
Imaging	Abdominal ultrasound; Intima-media thickness; Heart ultrasound; Fundoscopic examination by an ophthalmologist.	Elastography (liver and kidney).
Functional diagnostics	ECG; 24-h blood-pressure measurement; Oral glucose tolerance test with insulin levels; Pulse wave velocity; Measurement of body composition.	

**Table 2:** The integral model of cardiovascular disease prevention by age group.

Age (Years)/Risk Factor	Less Than 1 Year	1–4	5–8	9–11	12–17	18–21
Family history of early cardiovascular event		Family history given by parents: early cardiovascular events in close family (men ≤ 55 years, women ≤ 65 years)	Continuing history			Continuing history provided by the patient
Tobacco exposure	Advice on smoking indoors, help with cessationof smoking in parents	Continuing anti-smoking advice	Active anti-smoking advice to the child	Determination of smoking status in child, advice, helping with cessation of smoking		
Diet	Promoting breastfeeding, introduction of healthy food	Introduction of healthy food, counselling against sweet beverages and sweets			Determination of diet, encouraging healthy diet, diet counselling	
Growth, overweight, obesity	Counselling about appropriate growth, height/weight growth charts	Weight/growth charts, BMI determination after 2 years of age	Weight/growth charts, body mass BMI > 85th percentile: healthy lifestyle interventions; if no change in six months, counselling by a dietician is necessary, BMI > 95th percentile: obesity treatment			
Lipid profile	No lipid profile determined	Lipid profile determined if parents have dyslipidemia or other high-risk cardiovascular risk factors	Routine lipid profile at age of 5 years	Regular assessment of lipid profile if previously elevated; lipid profile determination if other cardiovascular risk factors; diet and possibly pharmacological management		
Blood pressure	Measurement in babies with kidney, urological, heart disease or if history of intensive care treatment	Annual measurement from 3 years of age, evaluation with percentile charts	Annual measurement, evaluation with percentile charts, exclusion of secondary hypertension, pharmacological treatment if very high values, unsuccessful non-pharmacological management, or presence of left heart hypertrophy			
Physical activity	Promotion of regular physical activity; no sedentary lifestyle (e.g., watching television) under the age of 2	Promotion of child’s active play, limitation of sedentary activities to less than two hours daily, no television or computer in the bedroom	History of physical activity, physical activity > one hour per day, sedentary activities < two hours per day			Discussion about the importance of healthy lifestyle with promotion of physical activity and limitation of sedentary lifestyle
Diabetes mellitus	When indicated, blood-sugar measurement with oral glucose tolerance test and determination of insulin level; referral to endocrinologist					

## Data Availability

All data is available within the article.
